# Nonlinear Conditions for Ultradifferentiability

**DOI:** 10.1007/s12220-021-00718-w

**Published:** 2021-06-19

**Authors:** David Nicolas Nenning, Armin Rainer, Gerhard Schindl

**Affiliations:** Fakultät für Mathematik, Universität Wien, Oskar-Morgenstern-Platz 1, 1090 Wien, Austria

**Keywords:** Joris theorem, Division property, Ultradifferentiable classes, (Non-)Quasianalytic, Holomorphic approximation, Almost analytic extension, 26E10, 30E10, 32W05, 46E10, 46E25, 58C25

## Abstract

A remarkable theorem of Joris states that a function *f* is $C^\infty$ if two relatively prime powers of *f* are $C^\infty$. Recently, Thilliez showed that an analogous theorem holds in Denjoy–Carleman classes of Roumieu type. We prove that a division property, equivalent to Joris’s result, is valid in a wide variety of ultradifferentiable classes. Generally speaking, it holds in all dimensions for non-quasianalytic classes. In the quasianalytic case we have general validity in dimension one, but we also get validity in all dimensions for certain quasianalytic classes.

## Introduction

A remarkable Theorem of Joris [11, Théorèm 2] states: if $f:\mathbb {R}\rightarrow \mathbb {R}$ is a function and *p*, *q* are relatively prime positive integers, then

1 $$\begin{aligned} f^p,f^q \in C^\infty \implies f \in C^\infty . \end{aligned}$$

Since smoothness can be tested along smooth curves by a theorem of Boman [3], one immediately infers that the implication (1) holds on arbitrary open subsets of $\mathbb {R}^d$, $d \ge 1$, and on smooth manifolds. (On the other hand, the regularity of a *single* power of a function generally says nothing about the regularity of the function itself; e.g. $(\mathbf {1}_{\mathbb {Q}} - \mathbf {1}_{\mathbb {R}\backslash \mathbb {Q}})^2 = 1$, where $\mathbf {1}_A$ is the indicator function of a set *A*.)

It was soon realized that the statement also holds for complex valued functions and it led to the study of so-called pseudoimmersions [7, 12, 13, 19]. A simple proof based on ring theory was given by [1].

Only recently Thilliez [30] showed that Joris’s result carries over to Denjoy–Carleman classes of Roumieu type $\mathcal {E}^{\{M\}}$. These are ultradifferentiable classes of smooth functions defined by certain growth properties imposed upon the sequence of iterated derivatives in terms of a weight sequence *M* (which in view of the Cauchy estimates measures the deviation from analyticity).

By extracting the essence of Thilliez’s proof, we show in this paper that a broad variety of ultradifferentiable classes has a division property equivalent to Joris’s result. Let $\mathcal {S}$ be a subring (with multiplicative identity) of the ring of germs at $0 \in \mathbb {R}^d$ of complex valued $C^\infty$-functions. We say that $\mathcal {S}$ has the *division property* $(\mathscr {D})$ if for any function germ *f* at $0 \in \mathbb {R}^d$ we have

2 $$\begin{aligned} \big (j \in \mathbb {N}_{\ge 1},~ f^j,f^{j+1} \in \mathcal {S}\big ) \implies f \in \mathcal {S}. \end{aligned}$$

If $\mathcal {S}$ has property $(\mathscr {D})$, then Joris’s theorem holds in $\mathcal {S}$. Indeed, suppose that $p_1,p_2$ are relatively prime positive integers and $f^{p_1},f^{p_2} \in \mathcal {S}$. All integers $j \ge p_1p_2$ can be written $j=a_1p_1 + a_2 p_2$ for $a_1,a_2 \in \mathbb {N}$, see [11, p.270]. Hence, $f^j \in \mathcal {S}$ for all $j \ge p_1p_2$. Since two consecutive integers are relatively prime, also the converse holds.^1^

### Results

Let us give an overview of our results.

The rings of germs in *one dimension* $d=1$ of the following ultradifferentiable classes have property $(\mathscr {D})$:

- $\mathcal {E}^{[M]}$, Denjoy–Carleman class of Roumieu (Theorems 2.2 and 2.3) and Beurling type (Theorem 2.6),
- $\mathcal {E}^{[\omega ]}$, Braun–Meise–Taylor classes of Roumieu and Beurling type (Theorem 3.1),
- $\mathcal {E}^{[\mathfrak {M}]}$, ultradifferentiable classes defined by weight matrices of Roumieu and Beurling type (Theorem 4.2).

It is understood that certain minimal regularity properties of the weights are assumed (see Table 1) which in particular guarantee that the sets of germs are indeed rings. (Note that by convention $[\cdot ]$ stands for $\{\cdot \}$, i.e., Roumieu, as well as $(\cdot )$, i.e., Beurling.)

Interestingly, the proof in one dimension works for quasianalytic and non-quasianalytic classes alike. But the tool used to reduce the multidimensional to the one-dimensional statement is only available in the non-quasianalytic Roumieu case ([15, 27]). The (multidimensional) Beurling case can often be reduced to the corresponding Roumieu case. Hence we obtain the following multidimensional non-quasianalytic results. The rings of germs in *all dimensions* *d* of the following ultradifferentiable classes have property $(\mathscr {D})$:

- $\mathcal {E}^{[M]}$, non-quasianalytic Denjoy–Carleman classes of Roumieu (Theorem 2.2) and Beurling type (Theorem 2.5),
- $\mathcal {E}^{[\omega ]}$, non-quasianalytic Braun–Meise–Taylor classes of Roumieu and Beurling type (Theorem 3.2),
- $\mathcal {E}^{\{\mathfrak {M}\}}$, non-quasianalytic ultradifferentiable classes defined by weight matrices of Roumieu type (Theorem 4.3).

For *quasianalytic* Denjoy–Carleman classes of Roumieu type $\mathcal {E}^{\{M\}}$ in *one* dimension the implication (2) follows from the stronger result, due to Thilliez [29], that $C^\infty$-solutions of a polynomial equation

3 $$\begin{aligned} z^n + a_1 z^{n-1} + \cdots + a_{n-1} z + a_n = 0, \end{aligned}$$

where the coefficients $a_j$ are germs at $0 \in \mathbb {R}$ of $\mathcal {E}^{\{M\}}$-functions, are of class $\mathcal {E}^{\{M\}}$ (under weak assumptions on *M*). This is false for non-quasianalytic classes. But it seems to be unknown whether, in the presence of quasianalyticity, it holds in higher dimensions. In fact, quasianalytic ultradifferentiability cannot be tested on quasianalytic curves (or lower dimensional plots) even if the function in question is known to be smooth ( [10, 20]).

Hence, we think that it is interesting that, combining our proof with a description of certain quasianalytic classes $\mathcal {E}^{\{M\}}$ as an intersection of suitable non-quasianalytic ones (due to [16]), we obtain that these quasianalytic classes have property $(\mathscr {D})$ in *all* dimensions (see Theorem 2.7 and also Remarks 3.3 and 4.4).

Since all considered regularity classes are local, the results for germs immediately give corresponding results for functions on open sets.

### Summary of the Results

We list in Table 1 the ultradifferentiable rings of germs known to have property $(\mathscr {D})$, together with the needed assumptions on the weights and the respective references. All germs are function germs at 0 in $\mathbb {R}^d$ for some dimension *d*. The dimension is added as a left subscript, e.g., ${_d}{\mathcal {E}}{^{[M]}}$ denotes the ring of germs at $0 \in \mathbb {R}^d$ of $\mathcal {E}^{[M]}$-functions. All notions will be defined below.

The Roumieu parts of the results in the first and the fifth row are due to Thilliez [30]; see Sects. 2.4, 2.5.

**Table 1:** Ultradifferentiable rings of germs having property $(\mathscr {D})$

Quasianalytic ring of germs		Reference
${_1}{\mathcal {E}}{^{[M]}}$	derivation closed	Theorems 2.3, 2.6
	*m* log-convex	
	$$m_k^{1/k} \rightarrow \infty $$ (Beurling)	
${_1}{\mathcal {E}}{^{[\omega ]}}$	$\omega$ concave	Theorem 3.1
${_1}{\mathcal {E}}{^{[\mathfrak {M}]}}$	[regular]	Theorem 4.2
	[moderate growth]	
${_d}{\mathcal {E}}{^{\{M\}}}$	intersectable	Theorem 2.7
	moderate growth	
Non-quasianalytic ring of germs		Reference
${_d}{\mathcal {E}}{^{[M]}}$	moderate growth	Theorems 2.2, 2.5
	*m* log-convex	
${_d}{\mathcal {E}}{^{[\omega ]}}$	$\omega$ concave	Theorem 3.2
${_1}{\mathcal {E}}{^{[\mathfrak {M}]}}$	[regular]	Theorem 4.2
	[moderate growth]	
${_d}{\mathcal {E}}{^{\{\mathfrak {M}\}}}$	$\{\text {regular}\}$	Theorem 4.3
	$$\{\text {moderate growth}\}$$	

We remark that non-quasianalytic Denjoy–Carleman classes $\mathcal {E}^{\{M\}}$, where the weight sequence *M* lacks moderate growth, do not have property $(\mathscr {D})$ in general; see [30, Remark 2.2.3]. The moderate growth condition is rather restrictive (e.g., it implies that the class $\mathcal {E}^{\{M\}}$ is contained in a Gevrey class). The consideration of the classes $\mathcal {E}^{[\omega ]}$ and $\mathcal {E}^{[\mathfrak {M}]}$ allows to overcome this restriction in the sense that the implication (2) holds under weaker moderate growth conditions.

### Strategy of the Proof

Thilliez’s proof of Joris’s theorem for $\mathcal {E}^{\{M\}}$ consists of the following two steps:

(i) The class $\mathcal {E}^{\{M\}}$ admits a description by holomorphic approximation which is based on a result of Dynkin [8] on almost analytic extensions and a related $\overline{\partial }$-problem.
(ii) If $f^j$, $f^{j+1}$ are of class $\mathcal {E}^{\{M\}}$ and $g_\varepsilon$, $h_\varepsilon$ are respective holomorphic approximations, the quotient $h_\varepsilon /g_\varepsilon$ is a naive candidate for a holomorphic approximation of *f*. In order to avoid small divisors one considers
  $$\begin{aligned} u_\varepsilon = \varphi _\varepsilon \frac{\overline{g}_\varepsilon h_\varepsilon }{\max \{|g_\varepsilon |,r_\varepsilon \}^2}, \end{aligned}$$

where $\varphi _\varepsilon$ is a suitable cutoff function and $r_\varepsilon >0$. For good choices of $r_\varepsilon$ the function $u_\varepsilon$ has uniform bounds and is close to *f*. The solution of a $\overline{\partial }$-problem is used to modify $u_\varepsilon$ in order to obtain a holomorphic approximation of *f*. By step (i) we may conclude that *f* belongs to $\mathcal {E}^{\{M\}}$.

Following the same strategy, we will work with weight matrices $\mathfrak {M}$, since they provide a framework for ultradifferentiability (Sect. 4) which encompasses Denjoy–Carleman classes (Sect. 2) and Braun–Meise–Taylor classes (Sect. 3). In Sect. 5 we prove a general characterization result by holomorphic approximation for $\mathcal {E}^{[\mathfrak {M}]}$ (Theorem 5.3) which extends step (i); it builds on the description by almost analytic extension presented in our recent paper [9]. Then we execute a version of step (ii) under a quite minimal set of assumptions, see Lemma 6.1. It enables us to easily deduce the main results in Sect. 6.

## Denjoy–Carleman Classes Have Property $(\mathscr {D})$

### Weight Sequences and Denjoy–Carleman Classes

Let $\mu =(\mu _k)$ be a positive increasing (i.e. $\mu _k \le \mu _{k+1}$) sequence with $\mu _0=1$. We define a sequence *M* by setting $M_k:= \mu _1 \cdots \mu _k$, $M_0 := 1$, and a sequence *m* by $m_k:=\frac{M_k}{k!}$. Clearly, $\mu$ uniquely determines *M* and *m*, and vice versa. In analogy we shall use sequences $N \leftrightarrow n \leftrightarrow \nu$, $L \leftrightarrow \ell \leftrightarrow \lambda$, etc.

That $\mu$ is increasing means that *M* is *log-convex*, i.e., $\log M$ is convex or, equivalently, $M_k^2\le M_{k-1}M_{k+1}$ for all *k*. If in addition $M_k^{1/k} \rightarrow \infty$, we say that *M* is a *weight sequence*.

Sometimes we will make the stronger assumption that *m* is log-convex.

For $\sigma > 0$ and open $U \subseteq \mathbb {R}^d$, one defines the Banach space

$$\begin{aligned} \mathcal {B}^M_\sigma (U) :=\Big \{ f \in C^\infty (U):~ \Vert f\Vert _{\sigma ,U}^M:= \sup _{x \in U,\, \alpha \in \mathbb {N}^d} \frac{|\partial ^\alpha f (x)|}{\sigma ^{|\alpha |}M_{|\alpha |}}<\infty \Big \} \end{aligned}$$

and the (local) *Denjoy–Carleman classes of Roumieu type*

$$\begin{aligned} \mathcal {E}^{\{M\}}(U):= \text {proj}_{V \Subset U } \text {ind}_{\sigma > 0} \mathcal {B}^{M}_\sigma (V). \end{aligned}$$

For later reference we also consider the global class $\mathcal {B}^{\{M\}}(V) := \text {ind}_{\sigma > 0} \mathcal {B}^{M}_\sigma (V)$. Replacing the existential quantifier for $\sigma$ by a universal quantifier, we find the *Denjoy–Carleman classes of Beurling type*

$$\begin{aligned} \mathcal {E}^{(M)}(U):= \text {proj}_{V \Subset U } \text {proj}_{\sigma > 0} \mathcal {B}^{M}_\sigma (V) \end{aligned}$$

and $\mathcal {B}^{(M)}(V) := \text {proj}_{\sigma > 0} \mathcal {B}^{M}_\sigma (V)$. We use the notation $\mathcal {E}^{[M]}$ for both $\mathcal {E}^{\{M\}}$ and $\mathcal {E}^{(M)}$, similarly for $\mathcal {B}^{[M]}$, etc.

For positive sequences *M*, *N*, we write $M \preccurlyeq N$ if $\sup _{k \in \mathbb {N}} \big (\frac{M_k}{N_k}\big )^{1/k}<\infty$ and $M \lhd N$ if $\lim _{k \rightarrow \infty } \big (\frac{M_k}{N_k}\big )^{1/k} = 0$. We have (cf. [23, Proposition 2.12])

$$\begin{aligned} M \preccurlyeq N \quad&\Leftrightarrow \quad \mathcal {E}^{[M]}(U) \subseteq \mathcal {E}^{[N]}(U),\\ M \lhd N \quad&\Leftrightarrow \quad \mathcal {E}^{\{M\}}(U) \subseteq \mathcal {E}^{(N)}(U), \end{aligned}$$

where for “$\Leftarrow$” one has to assume that *M* is a weight sequence. Note that $\mathcal {E}^{\{(k!)\}}(U)$ coincides with $C^\omega (U)$ and so the class of real analytic functions is contained in $\mathcal {E}^{(M)}$($\subseteq \mathcal {E}^{\{M\}}$) if and only if $m_k^{1/k} \rightarrow \infty$.

Log-convexity of *M* implies that $\mathcal {E}^{[M]}(U)$ is closed under pointwise multiplication of functions. Additional regularity properties for *M* endow $\mathcal {E}^{[M]}(U)$ with additional structure, e.g., log-convexity of *m* implies closedness under composition of functions. A crucial assumption in [30] is *moderate growth* of *M*, which reads as follows

4 $$\begin{aligned} \exists C>0~\forall k,j \in \mathbb {N}: ~M_{k+j} \le C^{k+j}M_kM_j. \end{aligned}$$

It implies *derivation closedness*

5 $$\begin{aligned} \exists C>0~\forall k \in \mathbb {N}: ~M_{k+1}\le C^{k+1}M_k. \end{aligned}$$

The last property we need to mention is *non-quasianalyticity* of *M*, that is

6 $$\begin{aligned} \sum _{k=1}^\infty \frac{1}{\mu _k}< \infty , \quad \text { or equivalently }\quad \sum _{k=1}^\infty \frac{1}{M_k^{1/k}} < \infty . \end{aligned}$$

By the Denjoy–Carleman theorem, this condition is equivalent to the existence of non-trivial functions with compact support in $\mathcal {E}^{[M]}(U)$. It is well-known that non-quasianalyticity implies $m_k^{1/k}\rightarrow \infty$.

Let ${_d}{\mathcal {E}}{^{[M]}}$ denote the *ring of germs* at $0 \in \mathbb {R}^d$ of complex valued $\mathcal {E}^{[M]}$-functions; here we assume that *M* is a weight sequence in order to have a ring.

#### Remark 2.1

There is a slight mismatch between our notation (also used in [9]) and that of [30] (and [22]). We write $M_j=m_jj!$ for weight sequences, so our *m* corresponds to *M* in [30].

### Associated Functions

Let $m = (m_k)$ be a positive sequence with $m_0=1$ and $m_k^{1/k} \rightarrow \infty$. We define the function

7 $$\begin{aligned} h_{m}(t) := \inf _{k \in \mathbb {N}} m_k t^k, \quad \text { for } t > 0, \quad \text { and } \quad h_{m}(0):=0, \end{aligned}$$

which is is increasing, continuous on $[0,\infty )$, and positive for $t>0$. For large *t* we have $h_{m}(t) = 1$. Furthermore, we need

8 $$\begin{aligned} \overline{\Gamma }_{m}(t)&:= \min \{k : h_{m}(t) = m_k t^k\}, \quad t > 0, \end{aligned}$$

and, provided that $m_{k+1}/m_{k} \rightarrow \infty$,

9 $$\begin{aligned} \underline{\Gamma }_{m} (t)&:= \min \Big \{k : \frac{m_{k+1}}{m_k} \ge \frac{1}{t} \Big \}, \quad t > 0. \end{aligned}$$

We trivially have $\underline{\Gamma }_m \le \overline{\Gamma }_m$. If *m* is log-convex, then $\overline{\Gamma }_{m} = \underline{\Gamma }_{m}$.

We shall use these functions for $m_k = M_k/k!$, where *M* is a weight sequence satisfying $m_k^{1/k}\rightarrow \infty$. Then $m_{k+1}/m_{k}\rightarrow \infty$ (since $M_k^{1/k} \le \mu _k$ for all *k*).

### Regular Weight Sequences

A weight sequence *M* is said to be *regular* if $m_k^{1/k} \rightarrow \infty$, *M* is derivation closed, and there exists a constant $C\ge 1$ such that $\overline{\Gamma }_m(Ct) \le \underline{\Gamma }_m(t)$ for all $t>0$.

### Denjoy–Carleman Classes of Roumieu Type Have Property $(\mathscr {D})$

#### Theorem 2.2

(Non-quasianalytic ${_d}{\mathcal {E}}{^{\{M\}}}$) Let *M* be a non-quasianalytic regular weight sequence of moderate growth. Then, ${_d}{\mathcal {E}}{^{\{M\}}}$ has property $(\mathscr {D})$.

This is a special case of Theorem 4.3 below (cf. Sect. 4.5). It implies Thilliez’s result [30, Corollary 2.2.5].

A quasianalytic one-dimensional version follows from a stronger result in [29]:

#### Theorem 2.3

(Quasianalytic ${_1}{\mathcal {E}}{^{\{M\}}}$) Let *M* be a quasianalytic derivation closed weight sequence such that *m* is log-convex. Then ${_1}{\mathcal {E}}{^{\{M\}}}$ has property $(\mathscr {D})$.

### Denjoy–Carleman Classes of Beurling Type Have Property $(\mathscr {D})$

Let us deduce Beurling versions of Theorems 2.2 and 2.3. We use the following lemma based on [14, Lemma 6] and [9, Lemma 7.5].

#### Lemma 2.4

Let *L*, *M* be positive sequences satisfying $L \lhd M$. Suppose that *m* is log-convex and satisfies $m_k^{1/k} \rightarrow \infty$. Then there exists a weight sequence *S* such that *s* is log-convex, $s_k^{1/k} \rightarrow \infty$, and $L\le S \lhd M$. Additionally, we may assume:

(i) *S* has moderate growth, if *M* has moderate growth.
(ii) *S* is derivation closed, if *M* is derivation closed.
(iii) *S* is non-quasianalytic, if *M* is non-quasianalytic.

#### Proof

Only the supplements (ii) and (iii) were not already proved in [9, Lemma 7.5].

(ii) follows from the fact that a weight sequence *M* is derivation closed if and only if there is a constant $C\ge 1$ such that $M_k \le C^{k^2}$ for all *k*, see [17, 18]. Since *S* is a weight sequence and $S \lhd M$, also *S* is derivation closed, by this criterion.

(iii) It suffices to show that there exists a non-quasianalytic weight sequence *N* such that $L \le N \lhd M$. Then we apply the lemma to $N \lhd M$ and obtain a weight sequence *S* with $N \le S \lhd M$ having all desired properties.

Let us show the existence of *N*. By $L \lhd M$, we have $\beta _k := \sup _{p \ge k} \big ( \frac{L_p}{M_p} \big )^{1/p} \searrow 0$. Applying [6, Lemme 16] (see also [28, Lemma 4.1]) to $\beta _k$ and $\alpha _k = \gamma _k := \frac{1}{\mu _k}$, yields an increasing sequence $\delta = (\delta _k)$ such that

10 $$\begin{aligned}&\delta _k \rightarrow \infty , \end{aligned}$$

11 $$\begin{aligned}&\delta _k \beta _k \rightarrow 0, \end{aligned}$$

12 $$\begin{aligned}&\frac{\mu _k}{\delta _k} \text { is increasing}, \end{aligned}$$

13 $$\begin{aligned}&\sum _{k=1}^\infty \frac{\delta _k}{\mu _k} \le 8 \delta _1 \sum _{k=1}^\infty \frac{1}{\mu _k} < \infty . \end{aligned}$$

Then $N_k := \frac{\mu _1 \cdots \mu _k}{\delta _1 \cdots \delta _k}$ defines a non-quasianalytic weight sequence, by (12) and (13). (Note that $\nu _k = \frac{\mu _k}{\delta _k} \rightarrow \infty$ is equivalent to $N_k^{1/k} \rightarrow \infty$.) It satisfies $N \lhd M$ by (10). By (11), there is a constant $C>0$ such that $\delta _k \big ( \frac{L_k}{M_k} \big )^{1/k} \le C$ for all *k*. By the monotonicity of $\delta$, this leads to $L_k \le C^k \frac{M_k}{\delta _k^k} \le C^k \frac{M_k}{\delta _1 \cdots \delta _k} = C^k N_k$. After replacing $(N_k)$ by $(C^k N_k)$ we have $L \le N \lhd M$. $\square$

#### Theorem 2.5

(Non-quasianalytic ${_d}{\mathcal {E}}{^{(M)}}$) Let *M* be a non-quasianalytic weight sequence of moderate growth such that *m* is log-convex. Then ${_d}{\mathcal {E}}{^{(M)}}$ has property $(\mathscr {D})$.

#### Proof

Suppose that $g := f^j, h:= f^{j+1} \in {_d}{\mathcal {E}}{^{(M)}}$ for some positive integer *j*. Assume that representatives of these germs are defined in the neighborhood of the closure of some bounded 0-neighborhood *U*; we denote the representatives by the same symbols. Then, the sequence

14 $$\begin{aligned} L_k:= \max \Big \{ \sup _{|\alpha |= k, \,x\in U} |g^{(\alpha )}(x)|, \sup _{|\alpha |= k, \,x\in U} |h^{(\alpha )}(x)| \Big \} \end{aligned}$$

satisfies $L \lhd M$. By Lemma 2.4, there exists a weight sequence *S* satisfying the assumptions of Theorem 2.2 and $L \le S \lhd M$. Thus, $f \in {_d}{\mathcal {E}}{^{\{S\}}} \subseteq {_d}{\mathcal {E}}{^{(M)}}$. $\square$

#### Theorem 2.6

(Quasianalytic ${_1}{\mathcal {E}}{^{(M)}}$) Let *M* be a quasianalytic derivation closed weight sequence such that *m* is log-convex and $m_k^{1/k} \rightarrow \infty$. Then ${_1}{\mathcal {E}}{^{(M)}}$ has property $(\mathscr {D})$.

#### Proof

This follows from the proof of Theorem 2.3 in [29] (which also works in the Beurling case). Alternatively, we may infer it from Theorem 2.3 by a reduction argument based on Lemma 2.4 as in the proof of Theorem 2.5. $\square$

### A Multidimensional Quasianalytic Result

Let *M* be a weight sequence and consider the sequence space

$$\begin{aligned} \Lambda ^{\{M\}} := \Big \{ (c_k) \in \mathbb {C}^\mathbb {N}: \;\exists \rho >0 : \sup _{k \in \mathbb {N}} \frac{|c_k|}{\rho ^k M_k} < \infty \Big \}. \end{aligned}$$

We call a quasianalytic weight sequence *M* *intersectable* if

15 $$\begin{aligned} \Lambda ^{\{M\}} = \bigcap _{N \in \mathcal {L}(M)} \Lambda ^{\{N\}}, \end{aligned}$$

where $\mathcal {L}(M)$ is the collection of all non-quasianalytic weight sequences $N \ge M$ such that *n* is log-convex. The identity (15) carries over to respective function spaces, since $\mathcal {B}^{\{M\}}(U) = \big \{f \in C^\infty (U) : (\sup _{x \in U}\Vert f^{(k)}(x)\Vert _{L^k_{\text {sym}}}) \in \Lambda ^{\{M\}}\big \}$, where $f^{(k)}$ denotes the *k*-th order Fréchet derivative and $\Vert \cdot \Vert _{L^k_{\text {sym}}}$ the operator norm.

Note that a quasianalytic intersectable weight sequence *M* always satisfies $m_k^{1/k} \rightarrow \infty$; an argument is given in Remark 2.8 below.

#### Theorem 2.7

(Quasianalytic ${_d}{\mathcal {E}}{^{\{M\}}}$) Let *M* be a quasianalytic intersectable weight sequence of moderate growth. Then ${_d}{\mathcal {E}}{^{\{M\}}}$ has property $(\mathscr {D})$.

The proof of this result is given in Sect. 6.

#### Remark 2.8

In [16, Theorem 1.6] (inspired by [2]) a sufficient condition for intersectability was given. Let *M* be a quasianalytic weight sequence with $1 \le M_0<M_1$. Consider the sequence ${\check{M}}$ defined by

$$\begin{aligned} {\check{M}}_k:= M_k \prod _{j=1}^k \Big (1 - \frac{1}{M_j^{1/j}}\Big )^k, \quad {\check{M}}_0 := 1. \end{aligned}$$

If ${\check{m}}$ is log-convex, then *M* is intersectable.

Not every quasianalytic weight sequence is intersectable, for instance,

$$\begin{aligned} \Lambda ^{\{(k!)\}} \ne \bigcap _{N \in \mathcal {L}((k!))} \Lambda ^{\{N\}} = \Lambda ^{\{Q\}}, \quad \text { where } Q_k = (k\log (k+e)^k; \end{aligned}$$

see [16, Theorem 1.8] and [26]. Every quasianalytic intersectable weight sequence *M* must satisfy $\Lambda ^{\{Q\}} \subseteq \Lambda ^{\{M\}}$, and so $m_k^{1/k}$ tends to $\infty$ since clearly $q_k^{1/k}$ does.

A countable family $\mathbf{Q} = \{Q^n\}_{n\in \mathbb {N}_{\ge 1}}$ of quasianalytic intersectable weight sequences of moderate growth was constructed in [16, Theorem 1.9]:

$$\begin{aligned} Q^n_k = \big (k \log (k) \log (\log (k)) \cdots \log ^{[n]}(k)\big )^k, \quad \text { for } k \ge \exp ^{[n]}(1), \end{aligned}$$

where $\log ^{[n]}$ denotes the *n*-fold composition of $\log$; analogously for $\exp ^{[n]}$.

See also [27, Sect. 11] for a generalization of this concept.

## Braun–Meise–Taylor Classes Have Property $(\mathscr {D})$

### Weight Functions and Braun–Meise–Taylor Classes

A *weight function* is, by definition, a continuous increasing function $\omega :[0,\infty ) \rightarrow [0,\infty )$ such that

$$(\omega _1)$$
$$\omega (2t) = O (\omega (t))$$ as $$t \rightarrow \infty $$,
$$(\omega _2)$$
$$\omega (t) = o(t)$$ as $$t \rightarrow \infty $$,
$$(\omega _3)$$
$$\log (t) = o(\omega (t))$$ as $$t \rightarrow \infty $$,
$$(\omega _4)$$
$t \mapsto \omega (e^t)=:\varphi _\omega (t)$ is convex on $[0,\infty )$.

One may assume that $\omega |_{[0,1]} \equiv 0$ (without changing the associated classes $\mathcal {E}^{[\omega ]}$) which we shall tacitly do if convenient.

Let $U \subseteq \mathbb {R}^d$ be open and $\rho >0$. We associate the Banach space

$$\begin{aligned} \mathcal {B}^{\omega }_\rho (U):= \Big \{f \in C^\infty (U): \Vert f\Vert ^\omega _{\rho ,U}:= \sup _{x \in U,\, \alpha \in \mathbb {N}^d} \frac{|\partial ^\alpha f (x)|}{e^{\varphi _\omega ^* (\rho |\alpha |)/\rho }}<\infty \Big \}, \end{aligned}$$

where $\varphi _\omega ^*(s):= \sup _{t \ge 0} \{st - \varphi _\omega (t)\}$ is the Young conjugate of $\varphi _\omega$ (which is finite by $(\omega _3)$). Then the (local) *Braun–Meise–Taylor class of Roumieu type* is

$$\begin{aligned} \mathcal {E}^{\{\omega \}}(U):= \text {proj}_{V \Subset U} \text {ind}_{n \in \mathbb {N}} \mathcal {B}^\omega _n(V), \end{aligned}$$

and that of *Beurling type* is

$$\begin{aligned} \mathcal {E}^{(\omega )}(U):= \text {proj}_{V \Subset U} \text {proj}_{n \in \mathbb {N}} \mathcal {B}^\omega _{\frac{1}{n}}(V). \end{aligned}$$

Again, we use $\mathcal {E}^{[\omega ]}$ for $\mathcal {E}^{\{\omega \}}$ and $\mathcal {E}^{(\omega )}$, similarly for $\mathcal {B}^{[\omega ]}$ etc.

For two weight functions $\omega$, $\sigma$ we have (cf. [23, Corollary 5.17])

$$\begin{aligned} \sigma (t) = O(\omega (t)) \text { as } t\rightarrow \infty \quad&\Leftrightarrow \quad \mathcal {E}^{[\omega ]}(U) \subseteq \mathcal {E}^{[\sigma ]}(U),\\ \sigma (t) = o(\omega (t)) \text { as } t\rightarrow \infty \quad&\Leftrightarrow \quad \mathcal {E}^{\{\omega \}}(U) \subseteq \mathcal {E}^{(\sigma )}(U). \end{aligned}$$

We say that $\omega$ and $\sigma$ are *equivalent* if they generate the same classes, i.e., $\sigma (t) = O(\omega (t))$ and $\omega (t) = O(\sigma (t))$ as $t \rightarrow \infty$.

A weight function is said to be *non-quasianalytic* if

16 $$\begin{aligned} \int _1^\infty \frac{\omega (t)}{t^2}\,dt<\infty . \end{aligned}$$

This is the case if and only if $\mathcal {E}^{[\omega ]}(U)$ contains non-trivial functions of compact support (cf. [5] or [22]).

Let us emphasize that in this paper we treat condition $(\omega _2)$ as a general assumption for weight functions; it means that the Beurling class $\mathcal {E}^{(\omega )}$ contains the real analytic class. It is automatically satisfied if $\omega$ is non-quasianalytic.

Let ${_d}{\mathcal {E}}{^{[\omega ]}}$ denote the *ring of germs* at $0 \in \mathbb {R}^d$ of complex valued $\mathcal {E}^{[\omega ]}$-functions; note that $\mathcal {E}^{[\omega ]}$ is stable by multiplication of functions for any weight function $\omega$.

### The Associated Weight Matrix

Let $\omega$ be a weight function. Setting $\Omega ^x_k:= e^{\varphi _\omega ^* (xk)/x}$ defines a weight sequence $\Omega ^x$ for every $x>0$, where $\Omega ^x \le \Omega ^y$ if $x \le y$. Thus the collection ${\varvec{\Omega }}:=\{\Omega ^x\}_{x>0}$ is a weight matrix (in the sense of Sect. 4). Note that ${\varvec{\Omega }}$ satisfies a *mixed* moderate growth property, namely

17 $$\begin{aligned} \;\forall x>0 \;\forall j,k \in \mathbb {N}: \Omega ^x_{j+k} \le \Omega ^{2x}_j \Omega ^{2x}_k. \end{aligned}$$

The importance of the associated weight matrix ${\varvec{\Omega }}$ is that it encodes an equivalent topological description of the spaces $\mathcal {E}^{[\omega ]}(U)$ as unions or intersections of Denjoy–Carleman classes; see Sect. 4.5. All this can be found in [22].

### Braun–Meise–Taylor Classes Have Property $(\mathscr {D})$

#### Theorem 3.1

(${_1}{\mathcal {E}}{^{[\omega ]}}$) Let $\omega$ be a concave weight function. Then ${_1}{\mathcal {E}}{^{[\omega ]}}$ has property $(\mathscr {D})$.

Evidently, it suffices to assume that $\omega$ is equivalent to a concave weight function.

For the multidimensional analogue we additionally assume non-quasianalyticity.

#### Theorem 3.2

(Non-quasianalytic ${_d}{\mathcal {E}}{^{[\omega ]}}$) Let $\omega$ be a non-quasianalytic concave weight function. Then ${_d}{\mathcal {E}}{^{[\omega ]}}$ has property $(\mathscr {D})$.

Theorems 3.1, 3.2 are corollaries of Theorems 4.2 and 4.3 below; for the proofs see Sect. 6.

#### Remark 3.3

Every weight sequence *M* in the family $\mathbf{Q}$ mentioned at the end of Remark 2.8 satisfies

$$\begin{aligned} \liminf _{k \rightarrow \infty } \frac{\mu _{a k}}{\mu _k} > 1 \end{aligned}$$

for some positive integer *a*. Hence there is a quasianalytic weight function $\omega _M$ (take, e.g., $\omega _M(t) := - \log h_M(1/t)$) such that ${_d}{\mathcal {E}}{^{[\omega _M]}} = {_d}{\mathcal {E}}{^{[M]}}$, by [4, Theorem 14]. So for all $M \in \mathbf{Q}$, the quasianalytic ring ${_d}{\mathcal {E}}{^{\{\omega _M\}}}$ has property $(\mathscr {D})$.

## The Most General Version of the Theorem

Let us formulate the main theorems in the most general setting available. The conditions we put on abstract weight matrices are tailored in such a way that weight matrices associated with weight functions are contained as special classes.

### Weight Matrices and Ultradifferentiable Classes

A *weight matrix* $\mathfrak {M}$ is, by definition, a family of weight sequences which is totally ordered with respect to the pointwise order relation on sequences, i.e.,

- $$\mathfrak {M}\subseteq \mathbb {R}^\mathbb {N}$$,
- each $M \in \mathfrak {M}$ is a weight sequence in the sense of Sect. 2.1,
- for all $M, N \in \mathfrak {M}$ we have $M \le N$ or $M \ge N$.

Let $U \subseteq \mathbb {R}^d$ be open. Given a weight matrix $\mathfrak {M}$, we define global classes

18 $$\begin{aligned} \mathcal {B}^{\{\mathfrak {M}\}}(U)&:= \text {ind}_{ M \in \mathfrak {M}} \mathcal {B}^{\{ M\}}(U), \end{aligned}$$

19 $$\begin{aligned} \mathcal {B}^{(\mathfrak {M})}(U)&:= \text {proj}_{ M \in \mathfrak {M}} \mathcal {B}^{( M)}(U). \end{aligned}$$

The limits in (18) and (19) can always be assumed countable, as is shown in [9, Lemma 2.5]. Writing $[\cdot ]$ for $\{\cdot \}$ and $(\cdot )$, the local classes are defined by

$$\begin{aligned} \mathcal {E}^{[\mathfrak {M}]}(U) := \text {proj}_{V \Subset U} \mathcal {B}^{[\mathfrak {M}]}(V). \end{aligned}$$

Let ${_d}{\mathcal {E}}{^{[\mathfrak {M}]}}$ denote the *ring of germs* at $0 \in \mathbb {R}^d$ of complex valued $\mathcal {E}^{[\mathfrak {M}]}$-functions; notice that $\mathcal {E}^{[\mathfrak {M}]}$ is stable by multiplication of functions, since each $M \in \mathfrak {M}$ is a weight sequence.

### Regular Weight Matrices

A weight matrix $\mathfrak {M}$ satisfying

- $$m_k^{1/k} \rightarrow \infty $$ for all $$ M \in \mathfrak {M}$$

is called $\{\text { regular}\}$ or *R-regular* (for Roumieu) if

- $$\forall M \in \mathfrak {M}\;\exists N \in \mathfrak {M}\;\exists C\ge 1 \;\forall j \in \mathbb {N}: M_{j+1} \le C^{j+1} N_j$$,
- $$\forall M \in \mathfrak {M}\;\exists N \in \mathfrak {M}\;\exists C\ge 1 \;\forall t>0 : \overline{\Gamma }_{ n}(Ct) \le \underline{\Gamma }_{ m}(t)$$,

and $(\text { regular})$ or *B-regular* (for Beurling) if

- $$\forall M \in \mathfrak {M}\;\exists N \in \mathfrak {M}\;\exists C \ge 1 \;\forall j \in \mathbb {N}: N_{j+1} \le C^j M_j$$,
- $$\forall M \in \mathfrak {M}\;\exists N \in \mathfrak {M}\;\exists C \ge 1 \;\forall t>0 : \overline{\Gamma }_{ m}(Ct) \le \underline{\Gamma }_{ n}(t)$$.

Moreover, $\mathfrak {M}$ is called *regular* if it is both R- and B-regular. By our convention, $[\text {regular}]$ stands for $\{\text {regular}\}$ (i.e. R-regular) in the Roumieu case and $(\text {regular})$ (i.e. B-regular) in the Beurling case.

### Almost Analytic Extensions

Let $h : (0,\infty ) \rightarrow (0,1]$ be an increasing continuous function which tends to 0 as $t \rightarrow 0$. Let $\rho >0$ and let $U \subseteq \mathbb {R}^d$ be a bounded open set. We say that a function $f : U \rightarrow \mathbb {C}$ admits an $(h,\rho )$-*almost analytic extension* if there is a function $F \in C^1_c(\mathbb {C}^d)$ and a constant $C\ge 1$ such that $F|_U = f$ and

$$\begin{aligned} |\overline{\partial }F(z)| \le C\, h ( \rho d(z,\overline{U})), \quad \text { for } z \in \mathbb {C}^d, \end{aligned}$$

where $\overline{\partial }F (z):= \sum _{j=1}^d\frac{\partial F(z)}{\partial \overline{z}_j} d\overline{z}_j$ and $d(z,\overline{U}) := \inf _{x \in \overline{U}} |z-x|$ denotes the distance of *z* to $\overline{U}$.

Let us apply this definition to the functions $h_m$ from (7), where $m_k = M_k/k!$ and *M* belongs to a given weight matrix $\mathfrak {M}$. Let $f : U \rightarrow \mathbb {C}$ be a function.

- *f* is called $\{\mathfrak {M}\}$-*almost analytically extendable* if it has an $(h_{ m},\rho )$-almost analytic extension for some $M \in \mathfrak {M}$ and some $\rho >0$.
- *f* is called $(\mathfrak {M})$-*almost analytically extendable* if, for all $M \in \mathfrak {M}$ and all $\rho >0$, there is an $(h_{ m},\rho )$-almost analytic extension of *f*.

#### Theorem 4.1

([9, Corollaries 3.3, 3.5]) Let $\mathfrak {M}$ be a [regular] weight matrix. Let $U \subseteq \mathbb {R}^d$ be open. Then $f \in \mathcal {E}^{[\mathfrak {M}]}(U)$ if and only if $f|_{V}$ is $[\mathfrak {M}]$-almost analytically extendable for each quasiconvex domain *V* relatively compact in *U*.

In Sect. 5 we shall use [9, Proposition 3.12], which is a key ingredient of the proof of Theorem 4.1.

### Weight Matrices of Moderate Growth

For positive sequences *M*, *N* set

20 $$\begin{aligned} \text {mg}(M,N) := \sup _{j,k \ge 0, \, j+k\ge 1}\left( \frac{M_{j+k}}{N_jN_k}\right) ^{1/(j+k)} \in (0,\infty ]. \end{aligned}$$

We say that a weight matrix $\mathfrak {M}$ has *R-moderate growth* or $\{\text {moderate growth}\}$ if

21 $$\begin{aligned} \;\forall M \in \mathfrak {M}\;\exists N \in \mathfrak {M}: \text {mg}(M,N)<\infty , \end{aligned}$$

and *B-moderate growth* or $(\text {moderate growth})$ if

22 $$\begin{aligned} \;\forall M \in \mathfrak {M}\;\exists N \in \mathfrak {M}: \text {mg}(N,M)<\infty . \end{aligned}$$

Again we say that $\mathfrak {M}$ has *moderate growth* if it has R- and B-moderate growth, and [moderate growth] stands for $\{\text {moderate growth}\}$ and $(\text {moderate growth})$, respectively.

### Denjoy–Carleman and Braun–Meise–Taylor classes in this Framework

By definition, Denjoy–Carleman classes are described by weight matrices $\mathfrak {M}=\{M\}$ consisting of a single weight sequence *M*. Observe that the weight matrix $\mathfrak {M}=\{M\}$ is regular if and only if the weight sequence *M* is regular, and it has moderate growth if and only if *M* has moderate growth.

Let $\omega$ be a weight function and let ${\varvec{\Omega }}$ be the associated weight matrix (cf. Sect. 3.2). Then, by [22, Corollaries 5.8 and 5.15], as locally convex spaces

$$\begin{aligned} \mathcal {E}^{[\omega ]}(U) = \mathcal {E}^{[{\varvec{\Omega }}]}(U), \end{aligned}$$

and $\mathcal {E}^{[\omega ]}(U) = \mathcal {E}^{[\Omega ]}(U)$ for all $\Omega \in {\varvec{\Omega }}$ if and only if

$$\begin{aligned} \exists H \ge 1 ~\forall t \ge 0 : 2 \omega (t) \le \omega (Ht) +H, \end{aligned}$$

which is in turn equivalent to the fact that some (equivalently each) $\Omega \in {\varvec{\Omega }}$ has moderate growth; see also [4].

The associated weight matrix ${\varvec{\Omega }}$ always has moderate growth, by (17). It is equivalent to a regular weight matrix $\mathfrak {S}$ (that means $\mathcal {E}^{[\omega ]} = \mathcal {E}^{[\mathfrak {S}]}$) if and only if $\omega$ is equivalent to a concave weight function. In fact, a $\mathcal {E}^{[\omega ]}$-version of the almost analytic extension theorem 4.1 holds if and only if $\omega$ is equivalent to a concave weight function; see [9, Theorem 4.8]. The weight matrix $\mathfrak {S}= \{S^x\}_{x>0}$ has the property that for each $x>0$ the sequence $s^x$ is log-convex and satisfies $\text {mg}(s^x,s^{2x}) =: H < \infty$ and thus $h_{s^x}(t) \le h_{s^{2x}}(Ht)^2$ for all $x,t>0$; see [25, Proposition 3].

### General Ultradifferentiable classes Have Property $(\mathscr {D})$

#### Theorem 4.2

(${_1}{\mathcal {E}}{^{[\mathfrak {M}]}}$) Let $\mathfrak {M}$ be a [regular] weight matrix of [moderate growth]. Then ${_1}{\mathcal {E}}{^{[\mathfrak {M}]}}$ has property $(\mathscr {D})$.

The proof is given in Sect. 6. It builds upon a characterization of the class $\mathcal {E}^{[\mathfrak {M}]}$ by holomorphic approximation; see Sect. 5.

We may infer a multidimensional result, since non-quasianalytic $\mathcal {E}^{\{\mathfrak {M}\}}$-regularity can be tested along curves; this useful tool is available in a satisfactory manner only in the non-quasianalytic Roumieu setting. We need two additional properties of the weight matrix:

23 $$\begin{aligned} \;\exists M \in \mathfrak {M}: ~ \sum _{k=0}^\infty \frac{1}{\mu _k}<\infty . \end{aligned}$$

which means that $\mathcal {E}^{\{\mathfrak {M}\}}$ admits non-trivial functions of compact support, and

24 $$\begin{aligned} \;\forall M \in \mathfrak {M}\;\exists N \in \mathfrak {M}: m^\circ \preccurlyeq n, \end{aligned}$$

where $m_k^\circ := \max \{m_j m_{\alpha _1}\cdots m_{\alpha _j}: \alpha _i \in \mathbb {N}_{>0},\, \alpha _1+\dots +\alpha _j = k\}$. Condition (24) is equivalent to composition closedness of $\mathcal {E}^{\{\mathfrak {M}\}}$ (which follows from the arguments in [22, Theorem 4.9]) and is satisfied by every R-regular weight matrix. Indeed, if $\mathfrak {M}$ is R-regular, then $\mathcal {E}^{\{\mathfrak {M}\}}$ has a description by almost analytic extension, by Theorem 4.1. It is easy to see (cf. [9, Proposition 1.1]) that the latter condition is preserved by composition of functions.

Under these assumptions, a function *f* defined on an open set $U \subseteq \mathbb {R}^d$ is of class $\mathcal {E}^{\{\mathfrak {M}\}}$ if and only if $f\circ c$ is of class $\mathcal {E}^{\{\mathfrak {M}\}}$ for all $\mathcal {E}^{\{\mathfrak {M}\}}$-curves in *U*; see [15, 16] and [27, Theorem 10.7.1].

#### Theorem 4.3

(Non-quasianalytic ${_d}{\mathcal {E}}{^{\{\mathfrak {M}\}}}$) Let $\mathfrak {M}$ be an R-regular weight matrix of R-moderate growth satisfying (23). Then ${_d}{\mathcal {E}}{^{\{\mathfrak {M}\}}}$ has property $(\mathscr {D})$.

#### Proof

This follows immediately from Theorem 4.2 and the above observations. $\square$

Note that Theorem 4.3 implies Theorem 2.2 as a special case.

#### Remark 4.4

The family $\mathbf{Q} = \{Q^n\}_{n \in \mathbb {N}_{\ge 1}}$ of quasianalytic intersectable weight sequences referred to at the end of Remark 2.8 actually is a regular weight matrix of moderate growth. The Roumieu class $\mathcal {E}^{\{\mathbf{Q}\}}$ is quasianalytic and, since Theorem 2.7 applies to every $M \in \mathbf{Q}$, we conclude that ${_d}{\mathcal {E}}{^{\{\mathbf{Q}\}}}$ has property $(\mathscr {D})$.

Note that there is no weight sequence *M* with $\mathcal {E}^{\{M\}} = \mathcal {E}^{\{\mathbf{Q}\}}$ and no weight function $\omega$ with $\mathcal {E}^{\{\omega \}} = \mathcal {E}^{\{\mathbf{Q}\}}$. This follows from the fact that $Q^{n} \le Q^{n+1} \not \preccurlyeq Q^{n}$, in analogy to the proof given in [22, Theorem 5.22]; see also Remark 5.25 there.

## Holomorphic Approximation of Functions in $\mathcal {E}^{[\mathfrak {M}]}$

In this section we prove a characterization of the class $\mathcal {E}^{[\mathfrak {M}]}$ (in dimension one) by holomorphic approximation. It generalizes [30, Proposition 3.3.2].

For notational convenience, we set $\Vert f\Vert _{A} := \sup _{z \in A} |f(z)|$ for any complex valued function *f*, where *A* is any set in the domain of *f*.

### Some Preparatory Observations

#### Lemma 5.1

Let *M*, *N* be weight sequences satisfying $m_k^{1/k} \rightarrow \infty$, $n_k^{1/k} \rightarrow \infty$, and $C:=\text {mg}(M,N)<\infty$. Then

25 $$\begin{aligned} h_m(t)&\le C^jn_j t^j h_n(Ct), \quad t>0, ~j \in \mathbb {N}, \end{aligned}$$

26 $$\begin{aligned} h_m(t)&\le h_n\Big (\frac{eC}{2}t\Big )^2, \quad t>0. \end{aligned}$$

#### Proof

Note that $\text {mg}(m,n) \le \text {mg}(M,N)$. Thus, for all $j \in \mathbb {N}$ and $t>0$,

$$\begin{aligned} h_m(t)&\le \inf _{k\ge 0} m_{k+j}t^{k+j} \le \inf _{k\ge 0} n_j n_k (Ct)^{k+j} = C^j n_j t^j h_n(Ct). \end{aligned}$$

For (26) we refer to [24, Lemma 3.13]. $\square$

For $\varepsilon >0$ let $\Omega _\varepsilon$ denote the interior of the ellipse in $\mathbb {C}$ with vertices $\pm \cosh (\varepsilon )$ and co-vertices $\pm i\sinh (\varepsilon )$. By $\mathcal {H}(\Omega _\varepsilon )$ we denote the space of holomorphic functions on $\Omega _\varepsilon$. The following lemma is a simple modification of [30, Lemma 3.2.4].

#### Lemma 5.2

Let *M*, *N* be two weight sequences satisfying $m_k^{1/k} \rightarrow \infty$, $n_k^{1/k} \rightarrow \infty$, and $C:=\text {mg}(M,N)<\infty$. Let $\varepsilon >0$. Let $g \in \mathcal {H}(\Omega _\varepsilon )\cap C^0(\overline{\Omega }_\varepsilon )$ and assume that there are constants $L,a_1,a_2>0$ such that

$$\begin{aligned} \Vert g\Vert _{\Omega _\varepsilon } \le L,\quad \Vert g\Vert _{[-1,1]} \le a_1h_m(a_2\varepsilon ). \end{aligned}$$

Then with $a_3:=\max \{a_1,L\}$ and $a_4:=eCa_2$ we have

$$\begin{aligned} \Vert g\Vert _{\Omega _{\varepsilon /2}} \le a_3 h_n(a_4 \varepsilon ). \end{aligned}$$

#### Proof

Let $f(z):=\frac{1}{a_1} g (\sin (\varepsilon z))$. Since $z \mapsto \sin (\varepsilon z)$ maps the horizontal strip $S:=\{z \in \mathbb {C}:~|{\text {Im}}(z)|<1\}$ to $\Omega _\varepsilon$, we get that $f \in \mathcal {H}(S) \cap C^0(\overline{S})$ is bounded by $K:= \max \{1,\frac{L}{a_1}\}$ on the whole of *S* and by $h_m(a_2\varepsilon )$ on $\mathbb {R}$. Thus an application of Hadamard’s three lines theorem gives

$$\begin{aligned} |f(z)|\le h_m(a_2\varepsilon )^{1-|{\text {Im}}(z)|}K^{|{\text {Im}}(z)|}, \quad z \in S. \end{aligned}$$

Since $h_m\le 1$ and every $w\in \Omega _{\varepsilon /2}$ can be written as $w = \sin (\varepsilon z)$ for some $w \in S$ with $|{\text {Im}}(w)|\le 1/2$, we obtain

$$\begin{aligned} |g(w)|\le a_1 (K h_m(a_2\varepsilon ))^{1/2}. \end{aligned}$$

The statement follows from (26). $\square$

### Condition $(\mathcal {P}_{[\mathfrak {M}]})$

Let $\mathfrak {M}$ be a weight matrix.

$$(\mathcal {P}_{\{\mathfrak {M}\}})$$
We say that a function $f : [-1,1] \rightarrow \mathbb {C}$ satisfies $(\mathcal {P}_{\{\mathfrak {M}\}})$ if there exist $M \in \mathfrak {M}$, constants $K,c_1,c_2>0$, and a family $(f_\varepsilon )_{0<\varepsilon \le \varepsilon _0}$ of functions $f_\varepsilon \in \mathcal {H}(\Omega _\varepsilon ) \cap C^0(\overline{\Omega }_\varepsilon )$ such that for all $0<\varepsilon \le \varepsilon _0$,
  27 $$\begin{aligned} \Vert f_\varepsilon \Vert _{\Omega _\varepsilon }&\le K, \end{aligned}$$
  28 $$\begin{aligned} \Vert f-f_\varepsilon \Vert _{[-1,1]}&\le c_1 h_m(c_2\varepsilon ). \end{aligned}$$
$$(\mathcal {P}_{(\mathfrak {M})})$$
We say that a function $f : [-1,1] \rightarrow \mathbb {C}$ satisfies $(\mathcal {P}_{(\mathfrak {M})})$ if for all $M \in \mathfrak {M}$ and all $c_2 > 0$ there exist constants $K,c_1>0$ and a family $(f_\varepsilon )_{0<\varepsilon \le \varepsilon _0}$ of functions $f_\varepsilon \in \mathcal {H}(\Omega _\varepsilon ) \cap C^0(\overline{\Omega }_\varepsilon )$ such that (27) and (28) hold for all $0<\varepsilon \le \varepsilon _0$.

Note that $(\mathcal {P}_{\{\mathfrak {M}\}})$ generalizes condition $(\mathcal {P}_{M})$ of [30].

### Description by Holomorphic Approximation

#### Theorem 5.3

(i) Let $M^{(i)}$, $1\le i \le 3$, be weight sequences with $(m^{(i)}_k)^{1/k} \rightarrow \infty$ and

29 $$\begin{aligned}&\;\exists B_1\ge 1 \;\forall t>0 : \overline{\Gamma }_{ m^{(2)}} (B_1 t) \le \underline{\Gamma }_{ m^{(1)}}(t), \end{aligned}$$

30 $$\begin{aligned}&\;\exists B_2\ge 1 \;\forall j \in \mathbb {N}: m^{(2)}_{j+1} \le B_2^{j+1}m^{(3)}_j. \end{aligned}$$

Then for each $f \in \mathcal {B}^{M^{(1)}}_{B_0}((-1,1))$ there exist positive constants $K,c_1,c_2$ and functions $f_\varepsilon \in \mathcal {H}(\Omega _\varepsilon )\cap C^0(\overline{\Omega }_\varepsilon )$ such that for all small $\varepsilon >0$

31 $$\begin{aligned} \Vert f_\varepsilon \Vert _{\Omega _\varepsilon }\le K, \quad \Vert f-f_\varepsilon \Vert _{[-1,1]}\le c_1 h_{m^{(3)}}(c_2\varepsilon ). \end{aligned}$$

The constants $K,c_1,c_2$ only depend on $B_i$, in particular, $c_2 = CB_0B_1$, where *C* is an absolute constant.

(ii) Let $N^{(i)}$, $1\le i \le 3$, be weight sequences with $(n^{(i)}_k)^{1/k} \rightarrow \infty$ and $\text {mg}(N^{(i)},N^{(i+1)})=D^{(i)}<\infty$. Let $f : [-1,1] \rightarrow \mathbb {C}$ be a function. Assume that there exist positive constants $K,c_1,c_2$ and functions $f_\varepsilon \in \mathcal {H}(\Omega _\varepsilon )\cap C^0(\overline{\Omega }_\varepsilon )$ such that for all small $\varepsilon >0$

32 $$\begin{aligned} \Vert f_\varepsilon \Vert _{\Omega _\varepsilon }\le K, \quad \Vert f-f_\varepsilon \Vert _{[-1,1]}\le c_1 h_{n^{(1)}}(c_2\varepsilon ). \end{aligned}$$

Then $f \in \mathcal {B}^{N^{(3)}}_\sigma ((-b,b))$ for every $b<1$, where $\sigma := \frac{2eD^{(1)} D^{(2)}c_2}{E(1-b)}$ and *E* is an absolute constant.

(iii) If $\mathfrak {M}$ is a [regular] weight matrix of [moderate growth], then

33 $$\begin{aligned} f \in \mathcal {B}^{[\mathfrak {M}]}((-1,1)) \Rightarrow f ~\text {satisfies}~(\mathcal {P}_{[\mathfrak {M}]})\Rightarrow f \in \mathcal {E}^{[\mathfrak {M}]}((-1,1)). \end{aligned}$$

Note that [regularity] of $\mathfrak {M}$ is needed for the first implication in (iii), [moderate growth] for the second. Item (iii) generalizes [30, Proposition 3.3.2].

#### Proof

We follow closely the proof of [30, Proposition 3.3.2].

(i) Let $f \in \mathcal {B}^{M^{(1)}}_{B_0}((-1,1))$. By [9, Proposition 3.12], there are constants $c_1,c_2>0$ and a function $F \in C^1_c(\mathbb {C})$ extending *f* such that

34 $$\begin{aligned} |\overline{\partial }F (z)| \le c_1 h_{m^{(3)}}(c_2 d(z,[-1,1])), \quad z \in \mathbb {C}. \end{aligned}$$

Note that $c_1=c_1(\Vert f\Vert ^{M^{(1)}}_{B_0}, B_0,B_1,B_2)$ and $c_2=12B_0B_1$. Then $w_\varepsilon := \overline{\partial }F \,\mathbf {1}_{\Omega _\varepsilon }$ satisfies

$$\begin{aligned} \Vert w_\varepsilon \Vert _{\mathbb {C}} \le c_1 h_{m^{(3)}}(Cc_2 \varepsilon ), \end{aligned}$$

where $C>0$ is an absolute constant such that $d(z,[-1,1]) \le C\varepsilon$ for $z \in \Omega _\varepsilon$. Moreover, the bounded continuous function

$$\begin{aligned} v_\varepsilon (z):= \frac{1}{2\pi i}\int _{\mathbb {C}} \frac{w_\varepsilon (\zeta )}{\zeta -z} \, d\zeta \wedge d \overline{\zeta }\end{aligned}$$

satisfies $\overline{\partial }v_\varepsilon = w_\varepsilon$ in the distributional sense, and we have

35 $$\begin{aligned} \Vert v_\varepsilon \Vert _{\mathbb {C}} \le c_1 h_{m^{(3)}}(C c_2 \varepsilon ). \end{aligned}$$

So $f_\varepsilon :=F- v_\varepsilon$ is holomorphic on $\Omega _\varepsilon$ and continuous on $\overline{\Omega }_\varepsilon$. The estimates (34) and (35) easily imply (31).

(ii) Let $f : [-1,1] \rightarrow \mathbb {C}$ satisfy (32). Consider $g_\varepsilon := f_\varepsilon - f_{2\varepsilon } \in \mathcal {H}(\Omega _{\varepsilon }) \cap C^0(\overline{\Omega }_{\varepsilon })$. Then $\Vert g_\varepsilon \Vert _{\Omega _\varepsilon } \le 2 K$ and $\Vert g_\varepsilon \Vert _{[-1,1]} \le 2c_1 h_{n^{(1)}}(2c_2\varepsilon )$. By Lemma 5.2,

$$\begin{aligned} \Vert g_\varepsilon \Vert _{\Omega _{\varepsilon /2}} \le \max \{c_1,2K\} \, h_{n^{(2)}}(2eD^{(1)}c_2 \varepsilon ). \end{aligned}$$

There exists a (universal) constant $E>0$ such that for any $b<1$ the closed disk with radius $E(1-b)\varepsilon$ around any $x \in [-b,b]$ is contained in $\Omega _{\varepsilon /2}$. The Cauchy estimates and (25) yield

$$\begin{aligned} \Vert g_\varepsilon ^{(j)}\Vert _{[-b,b]}&\le \frac{\max \{c_1,2K\}}{(E(1-b)\varepsilon )^{j}} j!\, h_{n^{(2)}}(2eD^{(1)}c_2\varepsilon ) \\&\le \max \{c_1,2K\} \Big (\frac{2 eD^{(1)} D^{(2)} c_2}{E(1-b)}\Big )^j N_j^{(3)}\, h_{n^{(3)}}(2eD^{(1)} D^{(2)}c_2\varepsilon ), \end{aligned}$$

which means $\Vert g_{\varepsilon }\Vert ^{N^{(3)}}_{\sigma ,[-b,b]} \le \max \{c_1,2K\}\, h_{n^{(3)}}(2eD^{(1)} D^{(2)}c_2\varepsilon )$ for $\sigma = \frac{2eD^{(1)} D^{(2)}c_2}{E(1-b)}$. Thus, if $\varepsilon _0>0$ is such that (32) holds for all $0<\varepsilon \le \varepsilon _0$, then

$$\begin{aligned} g := f_{\varepsilon _0} + \sum _{j = 1}^\infty g_{\varepsilon _0 2^{-j}} = f_{\varepsilon _0} + \sum _{j = 1}^\infty (f_{\varepsilon _0 2^{-j}} - f_{\varepsilon _02^{-j+1}}) \end{aligned}$$

converges absolutely in the Banach space $\mathcal {B}^{N^{(3)}}_\sigma ([-b,b])$. Clearly, for every $k \in \mathbb {N}$,

$$\begin{aligned} g = f_{\varepsilon _02^{-k}} + \sum _{j = k+1}^\infty (f_{\varepsilon _0 2^{-j}} - f_{\varepsilon _02^{-j+1}}), \end{aligned}$$

and $f = g$ on $[-b,b]$, since for $x \in [-b,b]$,

$$\begin{aligned} |f(x)-g(x)| \le |f(x)-f_{\varepsilon _02^{-k}}(x)| + \Big |\sum _{j = k+1}^\infty (f_{\varepsilon _0 2^{-j}}(x) - f_{\varepsilon _02^{-j+1}}(x))\Big | \end{aligned}$$

which tends to 0 as $k \rightarrow \infty$, by (32) and absolute convergence of the sum.

(iii) For the first implication in (33) in the Roumieu case, observe that for $f \in \mathcal {B}^{\{\mathfrak {M}\}}((-1,1))$ we have $f \in \mathcal {B}^{M^{(1)}}_{B_0}((-1,1))$ for some $B_0>0$ and $M^{(1)}\in \mathfrak {M}$. Then R-regularity of $\mathfrak {M}$ implies the existence of $M^{(2)}, M^{(3)} \in \mathfrak {M}$ such that (29) and (30) are satisfied. Thus (i) yields the desired holomorphic approximation.

In the Beurling case take any weight sequence $M^{(3)} \in \mathfrak {M}$. By B-regularity, we find $M^{(1)},~M^{(2)}$ such that (29) and (30) are satisfied. If $f \in \mathcal {B}^{(\mathfrak {M})}((-1,1))$, then $f \in \mathcal {B}^{M^{(1)}}_{B_0}((-1,1))$ for any $B_0>0$. Again (i) yields the desired holomorphic approximation (since $c_2 = C B_0 B_1$).

The second implication in (33) follows from (ii), since [moderate growth] of $\mathfrak {M}$ yields weight sequences $N^{(i)}$ fulfilling the assumptions of (ii). $\square$

## Proofs

We are now ready to prove the main results. We begin with a technical lemma in which we extract and slightly modify the essential arguments of [30, Sect. 4]. Its general formulation allows us to readily complete the pending proofs.

### A Technical Lemma

#### Lemma 6.1

Let *j* be a positive integer. Let $M^{(i)}$, $1 \le i \le \lceil \log _2(j(j+1))\rceil +7=:k$, be weight sequences satisfying $(m^{(i)}_\ell )^{1/\ell } \rightarrow \infty$ and

$$\begin{aligned} \;\exists B\ge 1 \;\forall t>0 : \overline{\Gamma }_{ m^{(2)}} (B t) \le \underline{\Gamma }_{ m^{(1)}}(t),\\ \text {mg}(M^{(i)},M^{(i+1)}) <\infty , \quad \text { for }2 \le i \le k-1. \end{aligned}$$

If $f:[-1,1] \rightarrow \mathbb {C}$ is such that $f^j,f^{j+1} \in \mathcal {B}^{[M^{(1)}]}((-1,1))$, then $f \in \mathcal {E}^{[M^{(k)}]}((-1,1))$.

#### Proof

Set $$g:=f^j$$ and $$h := f^{j+1}$$.

Let us begin with the Roumieu case. By Theorem 5.3(i), there exist families of holomorphic functions $(g_\varepsilon )$, $(h_\varepsilon )$ approximating *g*, *h*, respectively. More precisely, there exist positive constants $K,c_1,c_2$ and functions $g_\varepsilon ,h_\varepsilon \in \mathcal {H}(\Omega _\varepsilon ) \cap C^0(\overline{\Omega }_\varepsilon )$ such that, for all small $\varepsilon >0$,

36 $$\begin{aligned}&\max \{\Vert g_\varepsilon \Vert _{\Omega _\varepsilon },\Vert h_\varepsilon \Vert _{\Omega _\varepsilon }\} \le K, \end{aligned}$$

37 $$\begin{aligned}&\max \{\Vert g- g_\varepsilon \Vert _{[-1,1]}, \Vert h- h_\varepsilon \Vert _{[-1,1]}\} \le c_1h_{m^{(3)}}(c_2\varepsilon ). \end{aligned}$$

Then $$g_\varepsilon ^{j+1} - h_\varepsilon ^j \in \mathcal {H}(\Omega _\varepsilon ) \cap C^0(\overline{\Omega }_\varepsilon )$$ satisfies

$$\begin{aligned} |g_\varepsilon ^{j+1} - h_\varepsilon ^j|&\le |g_\varepsilon ^{j+1} - f^{j(j+1)}| + |f^{j(j+1)} - h_\varepsilon ^j| \\&\le (j+1) \max \{|g_\varepsilon |, |g|\}^j |g_\varepsilon - g| + j \max \{|h_\varepsilon |, |h|\}^{j-1} |h_\varepsilon - h| \\&\le c_3 h_{m^{(3)}}(c_2 \varepsilon ), \quad \text { on } [-1,1]. \end{aligned}$$

Thus Lemma 5.2 implies

38 $$\begin{aligned} \Vert h_\varepsilon ^{j} - g_\varepsilon ^{j+1}\Vert _{\Omega _{\varepsilon /2}} \le c_4 h_{m^{(4)}}(Ce c_2 \varepsilon ) =: \delta _\varepsilon , \end{aligned}$$

where *C* is chosen such that $C \ge \text {mg}(M^{(i)},M^{(i+1)})$ for all $2 \le i \le k-1$. (Here and below all constants $c_i$ are independent of $\varepsilon$.)

Consider the continuous function

$$\begin{aligned} u_\varepsilon := \varphi _{\varepsilon }\frac{\overline{g}_\varepsilon h_\varepsilon }{\max \{|g_\varepsilon |, r_\varepsilon \}^2}, \quad \text { with } r_\varepsilon := \delta _\varepsilon ^{\frac{1}{j+1}}, \end{aligned}$$

where $\varphi _\varepsilon$ is a smooth function compactly supported in $\Omega _\varepsilon$ and 1 on $\Omega _{\varepsilon /2}$. It coincides with $h_\varepsilon /g_\varepsilon$ in $\Omega _{\varepsilon /2} \cap \{|g_\varepsilon |>r_\varepsilon \}$, but is not holomorphic everywhere near $[-1,1]$. By taking $\varepsilon >0$ sufficiently small, we may assume that $\delta _\varepsilon \le r_\varepsilon \le 1$.

Lemmas 4.2.1 to 4.2.4 in [30] (which apply without change to our situation) lead to a holomorphic approximation $(f_\varepsilon )$ of *f* by solving a suitable $\overline{\partial }$-problem. Indeed, they show (using (36), (37), (38) and $h_{m^{(3)}}(t) \le h_{m^{(4)}}(eCt/2)$, by (26) since $h_{m^{(4)}} \le 1$) that

39 $$\begin{aligned} \Vert u_\varepsilon \Vert _{\Omega _{\varepsilon /2}}&\le (2K)^{1/j}, \end{aligned}$$

40 $$\begin{aligned} \Vert f- u_\varepsilon \Vert _{[-1,1]}&\le c_5 r_\varepsilon ^{1/j}, \end{aligned}$$

and that the bounded continuous function

$$\begin{aligned} v_\varepsilon (z) := \frac{1}{2\pi i}\int _{\Omega _{\varepsilon /2}} \frac{\overline{\partial }u_\varepsilon (\zeta )}{\zeta - z} \,d \zeta \wedge d\overline{\zeta }, \end{aligned}$$

which satisfies $\overline{\partial }v_\varepsilon = \overline{\partial }u_\varepsilon \mathbf {1}_{\Omega _{\varepsilon /2}}$ in the distributional sense in $\mathbb {C}$, fulfills

41 $$\begin{aligned} \Vert v_\varepsilon \Vert _{\Omega _{\varepsilon /2}} \le c_6 \delta _\varepsilon ^{1/s} \end{aligned}$$

where *s* is any real number with $s > j(j+1)$ (with $c_6$ depending on *s*).

Then $f_\varepsilon := u_{2\varepsilon }-v_{2\varepsilon }$ is holomorphic in $\Omega _\varepsilon$ and continuous on $\mathbb {C}$. By (39) and (41), $\Vert f_\varepsilon \Vert _{\Omega _{\varepsilon }}$ is uniformly bounded for all small $\varepsilon$, and by (40) and (41),

$$\begin{aligned} \Vert f-f_\varepsilon \Vert _{[-1,1]} \le c_{7}\delta _{2\varepsilon }^{1/s}. \end{aligned}$$

Put $s:=2^{k-6}=: 2^\ell$. A repeated application of (26) gives

$$\begin{aligned} h_{m^{(4)}}(t)^{1/s}\le h_{m^{(k-2)}}((Ce)^\ell t), \quad t>0. \end{aligned}$$

Thus, for all small $\varepsilon$,

42 $$\begin{aligned} \Vert f-f_\varepsilon \Vert _{[-1,1]} \le c_{7}\delta _{2\varepsilon }^{1/s}&= c_7 \big (c_4 h_{m^{(4)}}(2e Cc_2 \varepsilon )\big )^{1/s}\nonumber \\&\le c_7 c_4^{1/s} h_{m^{(k-2)}}(2 c_2(eC)^{\ell +1}\varepsilon ). \end{aligned}$$

So Theorem 5.3(ii) implies that $f \in \mathcal {E}^{\{M^{(k)}\}}((-1,1))$. This ends the proof in the Roumieu case.

For the Beurling we observe that, by assumption, we find for any (small) $c_2>0$ approximating sequences $(g_\varepsilon )$, $(h_\varepsilon )$ such that (36) and (37) are satisfied. Then follow the above proof until the end and notice that thus also in the final approximation (42) the constant $2c_2(eC)^{\ell +1}$ gets arbitrarily small as $c_2$ gets small. Again an application of Theorem 5.3 completes the proof. $\square$

### Proof of Theorem 4.2—${_1}{\mathcal {E}}{^{[\mathfrak {M}]}}$

We may assume that there is a positive integer *j* such that $g=f^j$, $h=f^{j+1}$ are elements of the ring ${_1}{\mathcal {E}}{^{[\mathfrak {M}]}}$. By composing with suitable linear reparameterizations, we may further assume that they are represented by elements of $\mathcal {B}^{[\mathfrak {M}]}((-1,1))$ which we denote by the same symbols.

In the Roumieu case, there exists $M^{(1)} \in \mathfrak {M}$ such that *g*, *h* are contained in $\mathcal {B}^{\{M^{(1)}\}}((-1,1))$ (by the linear order of $\mathfrak {M}$). By R-regularity and R-moderate growth of $\mathfrak {M}$, we find sequences $M^{(i)} \in \mathfrak {M}$ satisfying the assumptions of Lemma 6.1 which implies that $f \in \mathcal {E}^{\{M^{(k)}\}}((-1,1))$.

In the Beurling case, we fix an arbitrary $M \in \mathfrak {M}$ and we show that $f \in \mathcal {E}^{(M)}((-1,1))$. By B-regularity and B-moderate growth of $\mathfrak {M}$, we now get sequences $M^{(i)} \in \mathfrak {M}$ as required in Lemma 6.1, where $M^{(k)}=M$. By assumption, *g*, *h* are elements of $\mathcal {B}^{(M^{(1)})}((-1,1))$. Thus Lemma 6.1 gives $f \in \mathcal {E}^{(M)}((-1,1))$.

### Proof of Theorem 3.1—${_1}{\mathcal {E}}{^{[\omega ]}}$

This is an immediate corollary of Theorem 4.2 and the discussion in Sect. 4.5.

### Proof of Theorem 3.2—Non-quasianalytic ${_d}{\mathcal {E}}{^{[\omega ]}}$

We reduce the multidimensional result to the one-dimensional one.

In the Roumieu case ${_d}{\mathcal {E}}{^{\{\omega \}}}$, Theorem 3.2 is a simple corollary of Theorem 4.3; the weight matrix $\mathfrak {S}$ from Sect. 4.5 clearly satisfies (23) (since $\omega$ is non-quasianalytic).

The Beurling case ${_d}{\mathcal {E}}{^{(\omega )}}$ can be reduced to the Roumieu case by means of the following lemma (which is an adaptation of [21, Lemma 13]).

#### Lemma 6.2

Let $\omega$ be a non-quasianalytic concave weight function. Suppose that $f : [0,\infty ) \rightarrow [0,\infty )$ is any function satisfying $\omega (t) = o(f(t))$ as $t \rightarrow \infty$. Then there exists a non-quasianalytic concave weight function ${\tilde{\omega }}$ satisfying $\omega (t) = o({\tilde{\omega }}(t))$ and ${\tilde{\omega }}(t) = o(f(t))$ as $t \rightarrow \infty$.

#### Proof

It suffices to extract some constructions from the proof of [21, Lemma 13] (to which we refer for details). We may assume that $\omega$ is of class $C^1$. The condition $\omega (t) = o(t)$ as $t \rightarrow \infty$ implies that $\omega '(t) \searrow 0$ as $t \rightarrow \infty$.

Note that $\log (t) = o(\omega (t))$ and $\omega (t) = o(f(t))$ imply $f(t) \rightarrow \infty$ as $t \rightarrow \infty$. We define inductively three sequences $(x_n)$, $(y_n)$, and $(z_n)$ with $x_1=y_1=z_1 =0$, $x_2> 0$, and the following properties:

43 $$\begin{aligned}&\int _{x_n}^\infty \frac{\omega (t)}{1+t^2} dt \le \frac{1}{n^3}, \end{aligned}$$

44 $$\begin{aligned}&x_n > 2 y_{n-1} + n, \end{aligned}$$

45 $$\begin{aligned}&f(t) \ge n^2 \omega (t), \quad \text { for all } t\ge x_n, \end{aligned}$$

46 $$\begin{aligned}&\omega (x_n) \ge 2^{n-i} \omega (z_i), \quad 1 \le i \le n-1, \end{aligned}$$

47 $$\begin{aligned}&\omega '(y_n) = \frac{n-1}{n} \omega '(x_n), \end{aligned}$$

48 $$\begin{aligned}&\omega (z_n) = n \omega (y_n) - (n-1) \big (\omega (x_n) + (y_n-x_n) \omega '(x_n)\big ). \end{aligned}$$

Concavity of $\omega$ guarantees well-definedness of these conditions. Then

$$\begin{aligned} {\tilde{\omega }}(t) := {\left\{ \begin{array}{ll} (n-1) \big (\omega (x_n) + (t-x_n) \omega '(x_n) \big ) - \sum _{i=1}^{n-2} \omega (z_{i+1}) &{}\text { if } x_n \le t< y_n, \\ n \omega (t) - \sum _{i=1}^{n-1} \omega (z_{i+1}) &{}\text { if } y_n \le t < x_{n+1}, \end{array}\right. } \end{aligned}$$

defines a non-quasianalytic concave weight function of class $C^1$ satisfying

49 $$\begin{aligned} (n-2) \omega (t) \le {\tilde{\omega }}(t) \le n \omega (t), \quad \text { if } t \in [x_n,x_{n+1}) \text { and } n \ge 2. \end{aligned}$$

(Non-quasianalyticity follows from (43) and the second inequality in (49); cf. [21, Remark 14].) Together with (45) this implies that $\omega (t) = o({\tilde{\omega }}(t))$ and ${\tilde{\omega }}(t) = o(f(t))$ as $t \rightarrow \infty$. $\square$

Suppose that $g=f^j$, $h=f^{j+1}$ are representatives (of the corresponding germs) belonging to $\mathcal {B}^{(\omega )}(U)$ on some relatively compact 0-neighborhood *U* in $\mathbb {R}^d$ and consider the sequence $L_k$ defined in (14). Then for each integer $j \ge 1$ there exists $C_j>1$ such that

$$\begin{aligned} L_k \le C_j \exp (j \varphi ^*_\omega (k/j)), \quad \text { for all } k \in \mathbb {N}. \end{aligned}$$

Defining the function $\ell : [0,\infty ) \rightarrow \mathbb {R}$ by

$$\begin{aligned} \ell (t) := \log \max \{L_k,1\}, \quad \text { for } k \le t <k+1, \end{aligned}$$

and performing the subsequent steps in [21, Sect. 5], we find that $\ell \le \varphi ^*_{{\tilde{\omega }}} + \text {const}$, where ${\tilde{\omega }}$ is the weight function provided by Lemma 6.2. This means that *g*, *h* belong to $\mathcal {B}^{\{{\tilde{\omega }}\}}(U)$. Invoking Theorem 3.2 in the Roumieu case shows that $f \in {_d}{\mathcal {E}}{^{\{{\tilde{\omega }}\}}}$. Since $\omega (t) = o({\tilde{\omega }}(t))$ as $t \rightarrow \infty$ we may conclude that $f \in {_d}{\mathcal {E}}{^{(\omega )}}$.

### Proof of Theorem 2.7—Quasianalytic ${_d}{\mathcal {E}}{^{\{M\}}}$

The following lemma is a variant of [16, Theorem 1.6(3)].

#### Lemma 6.3

Let *M* be a quasianalytic intersectable weight sequence. Then:

(i) $$n_k^{1/k} \rightarrow \infty $$ for all $$N \in \mathcal {L}(M)$$.
(ii) If *M* has moderate growth, then for every $N \in \mathcal {L}(M)$ there exists $N' \in \mathcal {L}(M)$ such that $\text {mg}(N',N)<\infty$.

#### Proof

(i) is obvious, since $m_k^{1/k} \rightarrow \infty$ (cf. Sect. 2.8).

(ii) If *M* has moderate growth, then so has *m*. Set $C:= \text {mg}(m,m) <\infty$. For $N \in \mathcal {L}(M)$ we define $N'$ by $n'_k := C^k \min _{0 \le j \le k} n_j n_{k-j}$ or equivalently

$$\begin{aligned} n'_{2j} := C^{2j} \underline{\nu }_1^2 \underline{\nu }_2^2 \underline{\nu }_3^2 \cdots \underline{\nu }_j^2, \quad n'_{2j+1} := C^{2j+1} \underline{\nu }_1^2 \underline{\nu }_2^2 \underline{\nu }_3^2 \cdots \underline{\nu }_j^2 \underline{\nu }_{j+1}, \end{aligned}$$

where $\underline{\nu }_k := n_{k}/n_{k-1}$. Then clearly $\text {mg}(N',N)<\infty$. Since $\underline{\nu }_k$ is increasing, so is $\underline{\nu }'_k := n'_k/n'_{k-1}$, thus $n'$ is log-convex. Moreover,

$$\begin{aligned} n'_{2j} = C^{2j} n_j^2 \ge \frac{m_{2j}}{m_j^2} n_j^2 \ge m_{2j} \end{aligned}$$

and analogously $n'_{2j+1} = C^{2j+1} n_j n_{j+1} \ge m_{2j+1}$, so that $N'\ge M$. It remains to check that $N'$ is non-quasianalytic. Since $N'$ is log-convex, the sequence $(N'_k)^{1/k}$ is increasing and so it suffices to show that $\sum _j (N'_{2j})^{-1/(2j)}<\infty$. This is clear, since

$$\begin{aligned} (N'_{2j})^{1/(2j)} = ((2j)!\, C^{2j} n_j^2)^{1/(2j)} \ge \frac{2C}{e} j n_j^{1/j} \ge \frac{2C}{e} N_j^{1/j} \end{aligned}$$

and *N* is non-quasianalytic. $\square$

Let *M* be a quasianalytic intersectable weight sequence of moderate growth. Suppose that $g=f^j$, $h=f^{j+1}$ are elements of ${_d}{\mathcal {E}}{^{\{M\}}}$. Since *M* is intersectable, it suffices to show that $f \in {_d}{\mathcal {E}}{^{\{N\}}}$ for every $N \in \mathcal {L}(M)$. Fix such *N*. By Lemma 6.3, there exist $N^{(1)}, \dots , N^{(k)} \in \mathcal {L}(M)$ with $N^{(k)}=N$ such that the requirements of Lemma 6.1 are satisfied. (Note that $\mathcal {L}(M)$ is not a weight matrix in the sense of Sect. 4.1, because it is not totally ordered.)

Let *U* be an open 0-neighborhood in $\mathbb {R}^d$ on which we have $g, h \in \mathcal {E}^{\{M\}}(U)$ for representatives which are denoted by the same symbols. Take any curve $c \in \mathcal {E}^{\{N^{(1)}\}}(\mathbb {R}, U)$ with compact support. Then, by composition closedness of $\mathcal {E}^{\{N^{(1)}\}}$ as $n^{(1)}$ is log-convex, we have $g\circ c, h\circ c \in \mathcal {E}^{\{N^{(1)}\}}(\mathbb {R})$. After a linear change of variables, we may assume that $g\circ c, h\circ c \in \mathcal {B}^{\{N^{(1)}\}}((-1,1))$. Thus Lemma 6.1 yields that $f\circ c \in \mathcal {E}^{\{N\}}((-1,1))$. This implies that $f \in \mathcal {E}^{\{N\}}(U)$, by [16, Theorem 2.7].

## Footnotes

1 In an earlier version of the paper we considered the division property $\big (j \in \mathbb {N}_{\ge 1},~ g,q,fg \in \mathcal {S},~ f^j= qg\big ) \implies f \in \mathcal {S}$ which resulted from our wish to prove an ultradifferentiable version of the division theorem [13, Theorem 1]. But this property is equivalent to (2).
